# Thoracic Endovascular Aortic Repair for Blunt Thoracic Aortic Injury: Impact of Arch Type on the Rate of Type 1 Endoleak

**DOI:** 10.1016/j.atssr.2025.07.025

**Published:** 2025-08-28

**Authors:** Alexandra MacHugh, Fiorella Yep Mendizabal, Joseph DuBose, Pedro G. Teixeira, Karen M. Kim, George J. Arnaoutakis, Joshua C. Grimm, Aortic Trauma Foundation, Naveed Saquib, Anthony Estrera, Gustavo Oderich, Rana Afifi, Ezra Koh, Michelle McNutt, Dave Meyer, Binod Shrestha, Edmundo Dipasupil, Elina Quiroga, Nicolas Stafforini, Charles Fox, Rishi Kundi, Rami Gilani, David Turay, Xian Luo-Owen, Muhammad Aftab, Mohammed Al-Musawi, T. Brett Reece, Donald Jacobs, Rafael D. Malgor, Jeniann Yi, Erica Leith Mitchell, Martin Croce, Suzanne Moyer, Lalithapriya Jayakumar, Matthew J. Sideman, Christopher Mitromaras, Dimitrios Miserlis, Reshma Brahmbhatt, Lisa Bennett, Ernest Moore, Alexis Cralley, William Shutze, William Dockery, Laura Petrey, Timothy N. Phelps, Nicole Fox, Lisa Shea, John Berne, Ivan Puente, Mario F. Gomez, Dalier R. Mederos, Pedro Teixeira, Emily Leede, Frank Buchanan, Emilio Ramos, Marielle Ngoue, Sadia Ali, Davide Pacini, Luca Botta, Ciro Amodio, Tal Horer, David McGreevy, Ravi Rajani, Jaime Benarroch-Gampel, Christopher R. Ramos, Manuel Garcia-Toca, Kenji Inaba, Desmond Khor, Matt Smeds, Emad Zakhary, Michael Williams, Catherine Wittgen, Pierantonio Rimoldi, Ilenia D'Alessio, Nicola Monzio Compagnoni, Valerio Tolva, Neil Parry, Luc Dubois, John Bini, Karen Herzing, Marc Passman, Adam W. Beck, Mark Patterson, Ben Pearce, Emily Spangler, Jarrad Rowse, Danielle Sutzko, Andres Schanzer, Francesco Aiello, Edward Arous, Elias Arous, Douglas Jones, Dejah Judelson, Louis Messina, Tammy Nguyen, Jessica Simons, Robert Steppacher, James Haan, Kelly Lightwine, Vincent Riambau, Gaspar Mestres, Xavier Yugueros, Daniel Gil, Eduard Casajuana, Malachi Sheahan, Marie Unruh, Claudie Sheahan, Tapash Palit, Amit Chawla, Amadis Brooke, Melissa Donovan, Joe Giaimo, Bruce Torrance, Joao Rezende-Neto, Mario D’Oria, Sandro Lepidi, Peter Rossi, Viktor Reva, Trissa Babrowski, Ross Milner, Luka Pocivavsek, Christopher Skelly, Julie Dunn, Brittany Smoot, Sam Godin, Kevin Martin, Todd Vogel, Santi Trimarchi, Maurizio Domanin, Viviana Grassi, Matt Eagleton, Ali Azizzadeh, Bruce Gewertz, Galinos Barmparas, Donald Baril, Elizabeth Chou, Cassra Arbabi, NavYash Gupta, Sally Schonefeld, Theodore Teruya, Marc Schermerhorn, Mark Wyers, Allen Hamdan, Lars Stangenberg, Andy Lee, Patric Liang, Christina Marcaccio, Junaid Malek, Giovanni Ferrante, Emmanuel Nwachuka, Ralph Darlin, Xzabia Caliste, Benjamin B. Chang, Jeffrey C. Hnath, Paul B. Kreienberg, Alexander Kryszuk, Adriana Laser, Sean P. Roddy, Stephanie Saltzberg, Melissa Shah, Courtney Warner, Chin-Chin Yeh, Allison Berndtson, Asad Choudhry, Joseph Galante, Lee Seong, Seong K. Lee, Andrew Rosenthal, Rachele Solomon, Sergi Bellmunt, Robert Rhee, Susan Beale, Lewis E. Jacobson, Jamie Williams, Christopher Firek, Xiaofei Zhang, Alex Coronel, Megan Brenner, Zachary Wanken, Jahanzeb Kaikaus, Daniel Kindell, Richie Li, Erin McIntosh, Fatima Mustansir, Julia Suggs, Shirli Tay, Varun Dalmia, Ryan Wahidi, Muhammad Zeeshan, Henry Jefferson, Heather Grossman Verner, Cynthia Villalta, Rene Chidozie, R. Pulli, Rossella Di Domenico, Sara Speziali, Giorgio Turicchia, Mara Fanelli

**Affiliations:** 1 Department of Surgery and Perioperative Care, The University of Texas at Austin Dell Medical School, Austin, Texas; 2 Division of Vascular Surgery, The University of Texas at Austin Dell Medical School, Austin, Texas; 3 Division of Cardiothoracic Surgery, The University of Texas at Austin Dell Medical School, Austin, Texas

## Abstract

**Background:**

In recent years, thoracic endovascular aortic repair (TEVAR) has emerged as the standard of care for management of blunt thoracic aortic injury (BTAI), resulting in reduced rates of in-hospital mortality. We identified factors, including arch type, that may impact endoleaks among these patients.

**Methods:**

The Aortic Trauma Foundation registry was used to identify patients with BTAI managed with TEVAR from 2012 to 2023 for whom arch type was documented. Type 1 endoleak rates, graft specifications, lesion characteristics, demographics, and mechanism of injury were compared.

**Results:**

The analysis included 405 patients. Patients with type II and III arches were more likely to have an endoleak compared with those with type I arches (4.3% vs 7.7% vs. 1.1%, *P* = .019). Those with type 1 endoleak also had a higher maximum lesion diameter on average (31 mm vs 23 mm, *P* = .034). Left subclavian artery coverage conferred a higher endoleak rate (5.1% vs 1.2%, *P* = .019), particularly among those with type II or III arches (9.1%). Patients with endoleak did not have a higher mortality rate. Furthermore, there was no difference in rates of endoleak when comparing Society for Vascular Surgery grade, device dimensions, bovine anatomy, lesion length, sex, age, comorbidities, trauma center volume, Injury Severity Score, or mechanism.

**Conclusions:**

As endovascular therapies continue to be the mainstay in the surgical management of BTAI, identification of anatomic features that increase the risk of technical failure is critical. Especially in cases of zone 2 TEVAR, arch angulation appears to impact endoleak rate and could influence device selection.

**In Short**

▪ Patients with type II and III aortic arches are more likely to have a type 1 endoleak compared with those with type I arches.
▪ Left subclavian artery coverage confers a higher type 1 endoleak rate, particularly among those with type II or III arches.

Blunt thoracic aortic injury (BTAI) is a surgical emergency and remains the second most common cause of death in blunt trauma. Autopsy studies reveal that up to 80% of BTAI deaths occur before patients reach the hospital.^1^^,^^2^ For those who survive to the hospital, prompt repair is critical. Historically, open repair was used for management of these injuries, resulting in a high level of morbidity and mortality. The first reported case of endovascular repair for BTAI was in 1997.^3^ In the years since, thoracic endovascular aortic repair (TEVAR) has emerged as the standard of care for management of these injuries.

With increasing use of an endovascular paradigm, a significant decrease in morbidity and mortality has been observed.^4^ TEVAR has, however, introduced a new set of technical and anatomic considerations that influence efficacy and complication rates. Specifically, type 1 endoleak, characterized by incomplete sealing at the proximal end of the stent graft, is a common cause of graft failure. Understanding the relationship between aortic arch morphology and the incidence of type 1 endoleak is essential for refining endovascular strategies and optimizing device selection.

Aortic arch types—ranging from type I to type III—are defined by the relationship of the great vessel origins to the horizontal plane of the outer curvature of the arch.^5^ This classification system serves as a useful proxy for the degree of aortic arch angulation. This study analyzed the impact of aortic arch anatomy, among other factors, on the risk of type 1 endoleak in patients undergoing TEVAR for BTAI.

## Patients and Methods

The Aortic Trauma Foundation (ATF) registry was used to perform a retrospective analysis of patients with BTAI from January 2012 to October 2023. This is a prospective registry of BTAI diagnosis, treatment, and outcomes from 48 trauma centers worldwide. Information is gathered on demographics, comorbidities, lesion characteristics, anatomy, mechanism of injury, management, complications, and mortality.

**Figure 1:**
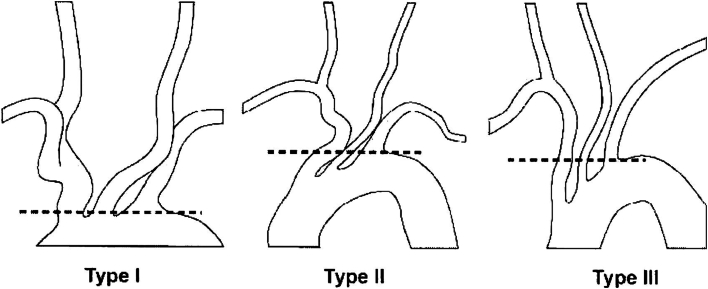
Aortic arch types I to III with dotted line indicating the horizontal plane of the outer curvature of the arch (provided by the Aortic Trauma Foundation).

In this study, only patients managed with TEVAR for whom arch type was documented were included for analysis. Arch type was identified by approximating the relationship of the great vessel origins to the outer curvature of the arch using computed tomography (CT) or angiographic imaging. The method of this categorization was left to the discretion of each recruiting institution (Figure 1).

The primary outcome was endoleak rate. All reported endoleaks in our study were type 1. The data collected were not further subcategorized into type 1a (proximal) or 1b (distal) endoleak. Virtually all type 1 endoleaks seen with TEVAR are type 1a, however, occurring at the proximal landing zone at the aortic arch. Secondary outcomes included in-hospital and aortic-related mortality. Categorical variables were compared with χ^2^ tests, and continuous variables were compared with analysis of variance tests using SAS statistical software (SAS Institute, Inc).

## Results

**Figure 2:**
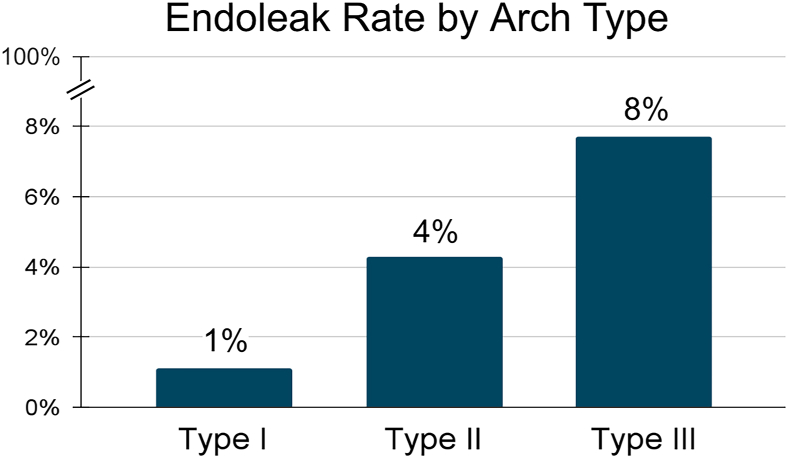
Endoleak rate by arch type.

Of >1200 patients with BTAI enrolled in the ATF registry, 405 patients met the aforementioned inclusion criteria. Among these patients, 273, 93, and 39, had type I, II, and III aortic arches, respectively. Patients with type II and III arches were significantly more likely to have an endoleak compared with those with type I arches (4.3% vs 7.7% vs 1.1%, *P* = .019) (Figure 2).

Failure of device delivery, failure of deployment, migration, maldeployment, and malposition by arch type were compared, and no differences were identified. Additionally, rates of reintervention were comparable among the arch types after TEVAR. There was also no significant difference in endoleak rate among those with bovine arch anatomy.

Coverage of the left subclavian artery for a zone 2 deployment was performed in 54% of the TEVARs in our sample. These were more likely to have an endoleak than those deployed more distally (5.1% vs 1.2%, *P* = .019). The rate of endoleak in zone 2 TEVARs with more aggressive arch angulation (ie, type II or III arches) was 9.1%.

In comparing device manufacturers, Gore endografts had fewer endoleaks than Medtronic, although this was not a statistically significant finding (0.94% vs 4.4%, *P* = .052). Relatively fewer Cook and Bolton grafts were used in the data set, and no endoleak difference was noted when including these manufacturers. We similarly assessed rates of endoleak by device length, as well as proximal and distal device diameters, and our analysis revealed no differences.

We investigated various lesion characteristics to evaluate for potential predictors of endoleak. We found no difference by Society for Vascular Surgeons grade, lesion length, percentage circumference of the aorta involved, or distance from the left subclavian artery. There was, however, a difference in relation to lesion location when comparing greater curvature, lesser curvature, and both curvatures involved. Endoleak rates were 6.8%, 2.3%, and 0.6%, respectively (*P* = .0275). Additionally, average maximum diameter was greater among those with endoleak (31 [SD, 16] mm) compared with those without (23 [SD, 11] mm) (Table 1).

**Table 1:** Anatomy, Lesion, and Device Characteristics

Variable	Type 1 Endoleak (n = 10)	No Endoleak (n = 395)	*P* Value
Arch type			.0198
Type I	3 (30)	270 (68)	
Type II	4 (40)	89 (23)	
Type III	3 (30)	36 (9)	
Bovine arch anatomy	3 (30)	78 (20)	.4456
Location			.0275
Greater curvature	5 (50)	74 (19)	
Lesser curvature	3 (30)	132 (33)	
Both	1 (10)	155 (39)	
Maximum diameter, mean (SD), mm	31 (16)	23 (11)	.0342
Left subclavian covered	7 (70)	131 (33)	.0196
Device manufacturer			.0522
Gore	2 (20)	212 (54)	
Medtronic	4 (40)	90 (23)	

Data are presented as n (%), unless indicated otherwise as mean (SD).

There was no difference in rates of endoleak when comparing sex, age, or comorbidities, including hypertension, smoking, or prior stroke. Furthermore, no difference was identified by mechanism, Injury Severity Score, trauma center volume, or after-hours repair. Patients with endoleak did not have a higher rate of in-hospital or aortic-related mortality (Table 2).

**Table 2:** Demographics and Endoleak Rate

Variable	Type 1 Endoleak (n = 10)	No Endoleak (n = 395)	*P* Value
Sex			.4925
Male	7 (70)	312 (79)	
Female	3 (30)	83 (21)	
Age, mean (SD), y	50 (18.6)	42 (17.6)	.1930
Injury Severity Score, mean (SD)	38 (22)	32 (14)	.2906
Hypertension	3 (30)	83 (21)	.4925
Smoker	1 (10)	81 (21)	.4142
Prior stroke	1 (10)	7 (2)	.0648
Trauma admits per year			.2102
<1000	0 (0)	49 (12)	
1000-2000	3 (30)	54 (14)	
2000-3000	5 (50)	135 (34)	
>3000	2 (20)	149 (38)	
Overnight repair 6 pm-6 am	3 (30)	141 (36)	.6889
In-hospital mortality	2 (20)	32 (8)	0.1802
Aortic-related mortality	1 (10)	7 (2)	0.0648

Data are presented as n (%), unless indicated otherwise as mean (SD).

## Comment

TEVAR has emerged as a pivotal intervention for treating BTAI by significantly improving patient outcomes, but it has also highlighted a set of technical considerations and distinct complications, including endoleak. Endoleak rate after TEVAR has been reported between 9% and 38%. Parmer and colleagues^6^ monitored 105 TEVAR patients and noted an endoleak rate of 29%, of which 40% were type 1, 35% were type 2, 20% were type 3, and 5% had >1 type of endoleak. Ninety percent of endoleaks were detected within the first month on routine postoperative CT scan. Fifty percent of the type 1 endoleaks were successfully repaired endovascularly.

Although some advocate for an endovascular approach to treat type 1a endoleak, an open or hybrid approach is often necessary if there is an insufficient proximal landing zone. Dun and colleagues^7^ monitored 23 patients who received operative intervention for type 1a endoleak after TEVAR and reported 15 were managed with total arch replacement with the frozen elephant trunk procedure, 2 with direct closure of the endoleak, 4 with hybrid aortic arch repair, 1 with arch debranching with TEVAR, and 1 with left subclavian artery-to-left carotid artery bypass with TEVAR. Despite the favorable risk profile seen with TEVAR, graft failure due to endoleak can still result in the need for high-risk salvage procedures.

With modern CT angiographic imaging, a great deal of preprocedural anatomic information is readily available. Arch type is one such key data point that can inform operative planning to help surgeons preempt complications that could ultimately require reintervention. It is accepted that with increased arch angulation comes an increased risk for type 1 endoleak; however, this study serves to quantify it.

Complete coverage of the left subclavian artery is performed in 50% to 70% of TEVARs. It is independently associated with several complications, including arm claudication and stroke, and is typically only performed in emergent situations to ensure an adequate proximal seal.^8^^,^^9^ Our analysis reveals that it may also increase the risk of a type 1 endoleak, which could, in the long-term, adversely impact outcomes. When it comes to arch morphology and proximal landing zone planning, graft choice may be influenced.

There are a myriad of considerations when choosing a graft, including surgeon experience, institutional preferences, patient characteristics, and anatomic factors. High-density grafts with increased radial force may be preferred for stability and resistance to migration, whereas flexible stent grafts are often selected when conformability is a priority. Our group is currently conducting a complementary ATF registry study that will provide an in-depth analysis of TEVAR device considerations and complications.

### Limitations

One limitation of this study is the subjective nature of arch type categorization. This was not quantified or standardized across recruiting institutions. Estimating the relationship of the great vessel origins to the line perpendicular to the greater curve is a practical way to quickly categorize and communicate aortic arch morphology, but it is not exact. Studies that quantify arch type do so by measuring the vertical distance from the level of the innominate artery origin to the top of the arch. The distance is <1 × left common carotid artery (LCCA) diameter in a type I arch, 1 to 2 × LCCA diameter in a type II arch, and >2 × LCCA diameter in a type III arch.^10^ Implementing this form of categorization would have removed a degree of subjectiveness from our study.

In addition to the inherent limitations as a retrospective study, this analysis also lacks information on long-term outcomes. Particularly in the setting of the quick and steep rise of endovascular management over the past 2 decades, monitoring long-term data will be an important future step. Furthermore, although the ATF data set has the strength of a large number of institutions involved with diverse patient populations and capabilities, the number of endoleaks reported is quite small (10), limiting the power of our statistical analyses.

### Conclusion

As endovascular therapies continue to be the mainstay in the surgical management of BTAI, identification of anatomic features that increase the risk of technical failure is critical. Especially in cases of zone 2 TEVAR, arch angulation appears to impact endoleak rate and could influence device selection.
