# Supplementary material for: Thoracic Endovascular Aortic Repair for Blunt Thoracic Aortic Injury: Impact of Arch Type on the Rate of Type 1 Endoleak
Source: Ann Thorac Surg Short Rep. 2025 Aug 28;4(1):12–6. doi: 10.1016/j.atssr.2025.07.025 (PMC13100764; doi:10.1016/j.atssr.2025.07.025)
Supplement: Supplementary Appendix [file mmc1.docx]

**Acknowledgements:**

We would like to acknowledge our collaborators at the Aortic Trauma Foundation: Naveed Saquib, Anthony Estrera, Gustavo Oderich, Rana Afifi, Ezra Koh, Michelle McNutt, Dave Meyer, Binod Shrestha, Edmundo Dipasupil, Elina Quiroga, Nicolas Stafforini, Charles Fox, Rishi Kundi, Rami Gilani, David Turay, Xian Luo-Owen, Muhammad Aftab, Mohammed Al-Musawi, T. Brett Reece, Donald Jacobs, Rafael D. Malgor, Jeniann Yi, Erica Leith Mitchell, Martin Croce, Suzanne Moyer, Lalithapriya Jayakumar, Matthew J Sideman, Christopher Mitromaras, Dimitrios Miserlis, Reshma Brahmbhatt, Lisa Bennett, Ernest Moore, Alexis Cralley, William Shutze, William Dockery, Laura Petrey, Timothy N Phelps, Nicole Fox, Lisa Shea, John Berne, Ivan Puente, Mario F Gomez, Dalier R Mederos, Pedro Teixeira, Emily Leede, Frank Buchanan, Emilio Ramos, Marielle Ngoue, Sadia Ali, Davide Pacini, Luca Botta, Ciro Amodio, Tal Horer, David McGreevy, Ravi Rajani, Jaime Benarroch-Gampel, Christopher R. Ramos, Manuel Garcia-Toca, Kenji Inaba, Desmond Khor, Matt Smeds, Emad Zakhary, Michael Williams, Catherine Wittgen, Pierantonio Rimoldi, Ilenia D'Alessio, Nicola Monzio Compagnoni, Valerio Tolva, Neil Parry, Luc Dubois, John Bini, Karen Herzing, Marc Passman, Adam W Beck, Mark Patterson, Ben Pearce, Emily Spangler, Jarrad Rowse, Danielle Sutzko, Andres Schanzer, Francesco Aiello, Edward Arous, Elias Arous, Douglas Jones, Dejah Judelson, Louis Messina, Tammy Nguyen, Jessica Simons, Robert Steppacher, James Haan, Kelly Lightwine, Vincent Riambau, Gaspar Mestres, Xavier Yugueros, Daniel Gil, Eduard Casajuana, Malachi Sheahan, Marie Unruh, Claudie Sheahan, Tapash Palit, Amit Chawla, Amadis Brooke, Melissa Donovan, Joe Giaimo, Bruce Torrance, Joao Rezende-Neto, Mario D’Oria, Sandro Lepidi, Peter Rossi, Viktor Reva, Trissa Babrowski, Ross Milner, Luka Pocivavsek, Christopher Skelly, Julie Dunn, Brittany Smoot, Sam Godin, Kevin Martin, Todd Vogel, Santi Trimarchi, Maurizio Domanin, Viviana Grassi, Matt Eagleton, Ali Azizzadeh, Bruce Gewertz, Galinos Barmparas, Donald Baril, Elizabeth Chou, Cassra Arbabi, NavYash Gupta, Sally Schonefeld, Theodore Teruya, Marc Schermerhorn, Mark Wyers, Allen Hamdan, Lars Stangenberg, Andy Lee, Patric Liang, Christina Marcaccio, Junaid Malek, Giovanni Ferrante, Emmanuel Nwachuka, Ralph Darlin, Xzabia Caliste, Benjamin B. Chang, Jeffrey C. Hnath, Paul B. Kreienberg, Alexander Kryszuk, Adriana Laser, Sean P. Roddy, Stephanie Saltzberg, Melissa Shah, Courtney Warner, Chin-Chin Yeh, Allison Berndtson, Asad Choudhry, Joseph Galante, Lee Seong, Seong K. Lee, Andrew Rosenthal, Rachele Solomon, Sergi Bellmunt, Robert Rhee, Susan Beale, Lewis E. Jacobson, Jamie Williams, Christopher Firek, Xiaofei Zhang, Alex Coronel, Megan Brenner, Zachary Wanken, Jahanzeb Kaikaus, Daniel Kindell, Richie Li, Erin McIntosh, Fatima Mustansir, Julia Suggs, Shirli Tay, Varun Dalmia, Ryan Wahidi, Muhammad Zeeshan, Henry Jefferson, Heather Grossman Verner, Cynthia Villalta, Rene Chidozie, R. Pulli, Rossella Di Domenico, Sara Speziali, Giorgio Turicchia, and Mara Fanelli.
